# Exploring the role of technology in human trafficking in Pakistan: A qualitative study of lived experiences of victims

**DOI:** 10.1371/journal.pone.0320088

**Published:** 2025-03-25

**Authors:** Zeeshan Khan, Mohammad Rahim Kamaluddin, Jamiah Manap, Surendran Rajaratnam, Masnizah Mohd, Ibrahim Maclean Chong, Farhan Navid Yousaf, Ratna Yunita Setiyani Subardjo

**Affiliations:** 1 Centre for Research in Psychology and Human Well-Being, Faculty of Social Sciences and Humanities, Universiti Kebangsaan Malaysia, Selangor, Malaysia; 2 Center for Cyber Security (CYBER), Faculty of Information Science and Technology, Universiti Kebangsaan Malaysia, Selangor, Malaysia; 3 Ministry of Human Resources Development, Federal Government Administration Center, Putrajaya, Malaysia; 4 Institute of Social and Cultural Studies, University of the Punjab, Lahore, Pakistan,; 5 Department of Psychology, Faculty of Economic Social Sciences and Humanities, University of 'Aisyiyah Yogyakarta, Indonesia; Shri Ramswaroop Memorial University, INDIA

## Abstract

With the ongoing digital transformation, the internet, mobile, social media and other computer technologies are being increasingly used in the targeting, recruitment, transportation and exploitation of human trafficking victims. The current study is the first of its kind which uses a qualitative method to comprehensively investigate the role of technology in the lived experiences of human trafficking victims in Pakistan. This qualitative study was carried out with a phenomenological approach in two provinces of Pakistan. Data was collected using in-depth interviews with 13 victims who were selected using purposive and snowball sampling methods. The data was then analyzed using Braun and Clarke’s thematic analysis approach. The data analysis results were divided into four main themes and ten sub-themes. The main themes are: Recruitment approaches, transportation process, exploitation process, and mental health consequences. The analysis show that traffickers extensively use technology across all phases of human trafficking. This study aims to support a range of relevant institutional stakeholders, including law enforcement agencies, legislative bodies, policymakers, civil society and SDG (2030) goals, in Pakistan and globally, in their efforts to address and enhance the learning and awareness level regarding related technologies in order to improve investigation in combating technological based human trafficking.

## Introduction

Human trafficking is a heinous issue and one of the most excruciating forms of human rights violations in the world [1–3]. The phenomenon of human trafficking refers to the recruitment, transportation, transfer, harboring, or receipt of an individual by using the techniques of fraud, force, and coercion for the purpose of sexual exploitation, forced labor, and payment benefits [4]. The Global Slavery Index 2023 documented that around 50 million people suffered in modern-day slavery in 2021, an increase of 10 million people compared to 2016 [5]. The majority of victims are primarily trafficked for sexual, labor exploitation, forced marriages, and financial gain [6–8]. Several reports document that every year millions of men, women, and children are trafficked across international borders and also within countries and near geographical areas [9,10]. Vulnerabilities to trafficking are shaped by poor financial conditions, unemployment, illiteracy, political insecurity, and natural disasters among others [9,11,12]. At the same time, victims who survived continue to suffer from severe mental, physical, and social consequences [1,10].

Advances in information and communication technologies have opened up new layers in the industry of human trafficking [13–15]. The use of the internet and social media technology has become one of the most important components of contemporary civilization [16,17]. According to Castells, the impact of new technology extends throughout the whole of human civilization [18]. As reported by Stata Research Department 2020, there are more than 3.5 billion social media users worldwide, and the number is expected to rise to over 4.41 billion users by 2025 [19–21]. The figure of 3.5 billion was reached by an increase of 2.86 billion in only four years, from 2017 to 2021 [22]. With the rise of social media technology and its users, the recruitment, transportation and exploitation of victims of human trafficking have also evolved. Internet, smartphones, Facebook, WhatsApp, Instagram and other Dark Web are found the most powerful tools in this process [13,23,24]. The traffickers use fake job advertisements, fake relations, fake profiles, and live video streaming to attract, recruit, transport, and exploit their victims [25,26]. Overall, the use of technology in human trafficking creates additional opportunities for traffickers to exploit their victims. It introduces new challenges for law enforcement agencies and their associated organizations that give rise to a potentially challenging issue for discussion [27,28].

Located in the Asian continent, Pakistan is the 5^th^ most populated country, with 241.49 million people, in the world [29]. Likewise, the country is also considered one of the top countries in the world when it comes to being the source and destination of human trafficking. The Global Slavery Index 2023 report claimed that around 2.3 million Pakistani people are living in modern-day slavery. In addition, Pakistan is among top 4 countries in Asia where 10.6 out of 1,000 population are living in modern-day slavery [30]. The traffickers in Pakistan use several mechanisms to attract, transport, and exploit the victims [31]. They also use different routes, borders, and communication strategies for executing their operations. Being one of the most densely populated countries, the number of social media users is continuously increasing in Pakistan [32,33]. According to the report of the Pakistan Telecommunication Authority (2022), more than 70 million people are active social media users in the country. Facebook, YouTube, and Instagram are the most popular and useable social media websites, where Facebook found the highest number of users 73.56% in 2022 [34,35]. The report on modern slavery in Pakistan 2019 indicated that the internet and mobile phones are widely used by traffickers for fake relationships, coercion, sale, purchase, and exploitation of Pakistani people [36].

Due to the hidden nature of human trafficking, the availability of comprehensive data is rather challenging [1,37]. Several researchers, academicians, and related organizations have yet try to explore and observe the link between technology and human trafficking [38,39]. In parallel, the role of technology in human trafficking in Pakistan has not yet been debated or explored academically with primary or secondary research data. For this purpose, this qualitative research study aims to fill the existing gap, and to contribute and add knowledge in the academic, policymaking and other spheres. Specifically, this study aims to examine the lived experiences of victims by exploring the role of technology in their victimization. Moreover, this study aims to provide evidence to a range of relevant institutional stakeholders, including law enforcement agencies, legislative bodies, policymakers, and civil society, both in Pakistan and globally. This evidence can then be incorporated in their efforts to address and enhance the learning and awareness levels regarding trafficking and associated technologies, and by extension, contribute to efforts of combatting human trafficking. In addition, this study will also contribute to the UN Sustainable Development Goals (SDG 2030), particularly 8.7, 5.2, 16.2, and 17.6 which deals with the elimination of human trafficking, exploitations, collaborations and technologies.

## Materials and methods

### Study area and location

One of the key steps in achieving a meaningful purpose of a research study is the selection of location [40]. The present research was carried out in two provinces of Pakistan, namely Punjab and Khyber Pakhtunkhwa. Approaching the victims is very challenging for a researcher under the certain circumstances, particularly, when the population of interest is hidden in nature [41]. Moreover, this study was an entirely new, emerging issue in the context of Pakistani society. Therefore, for reliability, validity, and accessibility, the researchers selected the victims from two provinces. These are the most densely populated provinces, which were documented as the main hot spot for human trafficking activities [42].

### Study design and research approaches

For this study, the researchers adopted the qualitative phenomenological approach with in-depth interviews to examine, comprehend, and investigate the lived experiences of victims about the role of technology in their victimizations. The phenomenological approach is primarily concerned with exploring the life stories and lived experiences of people under certain conditions [43,44]. The scientific writing of this study adhered with the outline of Consolidated Criteria for Reporting Qualitative Studies (COREQ) [45].

### Participants and sample size

For this study, the victim participants were selected using purposive and snowball sampling from two provinces of Pakistan based on the inclusion criteria: a) Aged 18 and above, b) victims recruited, transported or victimized using technology, c) willing to participate in the study and d) not diagnosed with any mental illness and exclusion criteria: a) Under 18 years of age b) victims recruited, transported or victimized without using technology, c) unwilling to participate and d) mentally unfit. When considering sample sizes, it was noticed that different studies had recommended different standards of sample size to be implemented in a qualitative study. Manson 2010 and Collins 2007 found seven sources that represented various methodologies for determining sample size in qualitative research, for instance Ethnography 30 to 50 interviews, Ethnoscience 30 to 60, Grounded theory methodology 20 to 30, Phenomenology 5 to 25 at the minimum 6 interviews, and all qualitative not less than 15 interviews.

Following these approaches, for this study, a total number of 13 victims were interviewed face-to-face within three months. Initially, security agencies and local community references assisted in reaching out to 29 victims through mobile phones. Of the 29 victims, only 17 were found recruited, transported, and exploited in human trafficking through using technological tools, and only 13 were agreed and participated in the interview. The others did not participate due to their unavailability and lack of interest. These victims were interviewed until no new emerging information was generated. This state is called saturation, and it typically occurs when a researcher receives no new information on the current topic and the participants repeat the same answers, leading the researcher to stop collecting more data [46,47]. In addition, there was no relationship between the researcher and the participants before conducting the study. The rapport was established with the participants through open communication and informed consent process. Every query the participants had concerning about the study was addressed and they were completely satisfied.

### Instruments and data collection

For this study, the researchers used an interview guide to understand the lived experiences of victims. An interview guide provides a framework to conduct a research interview with a more precise, detailed and rich understanding [48]. The researchers finalized the interview guide by conducting the content validity and pilot study interviews. The content validity was approved by a human trafficking expert from Pakistan. The interviews were conducted from November 4, 2023 until January 22, 2024.

All the interviews were carried out by the first author, he was born and raised in Pakistan and received training on qualitative research methodology before conducting the study. The first author became interested to study the impact of human trafficking on the victims in Pakistan while working as a researcher in communities focusing on socio-economic issues. Realizing that a number of people he came across either became victims or have family members or friends who became victims due to socio-economic pressure instilled the interest in him to understand the ease and impact of the use of technology in this activity. He is also doing his PhD research study on assessing the role of technology in human trafficking in Pakistan. In addition, this study team consists of both women and men of various academic ranking (i.e., Professor, Associate Professors and Senior Lecturer), from two different Universities, one in Pakistan. The team members also have expertise, with extensive research and practice experience in human trafficking, criminology, information communication technology and migration.

For the data collection, the first author travelled to each study participant’s home town and conducted interviews in locations which are safe and conducive, one that was preferred by the study participants. No non-participants were present during the interviews. Standardised interview procedures were employed to determine and reduce the possibility of interviewer bias in an effort to assure uniformity and neutrality in data collecting [49]. Before starting the interviews, each participant was informed about the role and academic affiliation of the researchers, the confidentiality of the data, and requested to acknowledge the informed consent form once they were content with the description of the study purpose, interview procedure, and the subsequent publishing of the findings. Each of the interviews was conducted only once without any subsequent sessions and these interviews were audio-recorded using voice recorder by the interviewer upon consent. Upon completion of the interviews, the researcher reflected on the key points discussed and summarized it to each of the participants to gauge their agreement on the points made by them. This reflection and summary allowed the participants to provide feedback and clarify their responses to the interviewer. The researcher further requested each participant on the findings feedback, but the participants show no interest for further follow-up due to the security concerns. Additionally, the researcher made field notes upon completion of each interview session to aid the data analysis process. Total duration of each interview was 60 to 90 minutes.

### Data analysis

The analysis of this study was carried out by using the thematic analysis manual method. For this purpose, the researchers followed the six different steps of Braun and Clarke’s thematic analysis approach [50]. After conducting the interviews, the first author followed the initial step of “familiarization with data” where the author transcribed and translated each of the interview recordings, then carefully read and reread the data to familiarize with it. In the second step of “generation initial code” initial codes were extracted after a careful and line-by-line review of the data. In the third step of “searching for themes among codes” the first author combined all codes and makes coherence with different themes and writes them down in one place. In the next “reviewing themes” step, the potential themes were developed from the initially generated codes and were reviewed repeatedly. In the fifth step, which is to “defining names and themes” the author finalized the themes by defining their names and generated the subthemes for further data analysis process. Subsequently, a workshop was organized among the co-authors to discuss and finalize the coded data and to evaluate the themes and sub-themes derived from the coding process. Finally, in the last step, all the data analysis procedures were rechecked by all of the authors, and results of the study were drafted. A total of four main themes and ten sub-themes were generated from the whole process of data analysis. The detail of the main themes and sub-themes is presented in the result section. In order to guarantee participants anonymity, confidentiality, and data security, all the interview files were saved on a private password-protected computer that was only accessible to the first author.

### Ethical approval

This research study was approved by the Research Ethics Committee, The National University of Malaysia, (Reference No. UKM PPI/111/8/JEP-2023-680). To uphold the ethical considerations, every participant was allowed to withdraw from the study at any moment without any reason.

## Results

Before proceeding to write the results of the thematic analysis, it is very important to showcase the demographic composition of each participant. Table 1 presents the demographic profile of the current study participants, where the majority, (n = 11) participants, were males and only (n = 2) were females. The age of the participants, on the other hand, ranges from 22 to 55 years, with a higher number of (n = 7) participants falling between the age of 22 and 25 years. More than half of (n = 8) participants were unmarried, with only (n = 4) being married. A majority of (n = 9) participants only possessed levels of academic qualifications, ranging from Primary to Intermediate levels of schooling, while only (n = 2) participants had a Master’s Degree. Likewise, more than half (n = 8) of the participants were living in very poor conditions and worked as laborers in different shops and agricultural lands. Several participants’ family monthly income fell within the range of 30 to 50 thousand Pakistani Rupee (PKR).

**Table 1:** Demographic profile of participants.

Participant	Gender	Age	Marital Status	Education	Occupation	Family monthly income PKR (1 US Doller = 280 PKR)
1	Male	31	Unmarried	Primary (5 levels)	Labor	Not mentioned
2	Male	21	Unmarried	Matric (10 levels)	Driver	Not mentioned
3	Male	30	Married	Uneducated	Labor	50 thousand
4	Male	55	Married	Primary	Retired from security agency	50 thousand
5	Male	36	Married	Master	Personal business	80 thousand to 100,000
6	Male	22	Unmarried	Middle (8 levels)	Farmer	30 to 50 thousand
7	Male	22	Unmarried	Intermediate (12 levels)	Labor	30 thousand
8	Male	25	Unmarried	Master	Teacher	50 thousand
9	Female	23	Married	Matric	Housewife	30 to 40 thousand
10	Female	25	Divorced	Middle	Housekeeper	30 thousand
11	Male	27	Unmarried	Bachelor	Unemployed	30 to 35 thousand
12	Male	24	Unmarried	Matric	Farmer	Not mentioned
13	Male	26	Unmarried	Intermediate	Labor	40 thousand

After the data collection and thematic analysis process, four main themes including the Recruitment Process, Transportation Process, Exploitation Process, and Mental Health Consequences were explored from the interviews. In addition, ten sub-themes were also developed from the four main themes. Table 2 shows the datils of the main themes and sub-themes of this study analysis.

**Table 2:** Details of main themes and sub-themes.

Main Themes	Sub-Themes
Recruitment approaches	Onset of victimization at a younger age Financial motivation of victims in the recruitment Participation of close companions in the recruitment The use of cyber platforms in the recruitment
Transportation process	Local transportation used as a means for victim’s transportation Utilization of cyber platforms in the process of transportation
Exploitation process	Receiving money as a main motive of victims’ exploitation Utilization of cyber platforms in the exploitation process
Mental health consequences	Adverse psychological implications on victims Adapting behavior towards social media

### Recruitment approaches

The first main theme that appears from the participants’ interviews is the recruitment approaches. Human trafficking is considered to be an organized crime. The traffickers have strong connections around the world. They target, compel, and recruit victims from different countries and regions and put them in exploitative conditions. For this purpose, the traffickers use advanced strategies and techniques to easily lure and recruit victims and then execute their purposes. Subsequently, the following four sub-themes are explored from the main theme.

#### Onset of victimization at a younger age.

It is essential to understand and evaluate the participant’s perspective regarding the particular moment where the accident occurred or began. Likewise, the victim participants of this study also shared their experiences about the consent of their victimization. The analysis of this study concluded that several (n = 8) participants were victimized at a younger age; between 19 and 25 years. The participants said:

“I was victimized at the end of 2021; I was 19 years old” (Participant 2).

“I was 20 years old when the agent did fraud with me” (Participant 6).

“At that time, my age was 23 years, when I was sexually exploited” (Participant 10).

#### Financial motivation of victims in the recruitment.

The crime of human trafficking upholds several factors that contribute to the recruitment of victims. These factors may include social, economic, political, or similar aspects. Nevertheless, this study’s participants also identified the factors that were crucial to their recruitment. Concerning this, more than half (n = 10) of the participants accepted that financial motivation was the main factor behind their recruitment.

“I am retired from as a sepoy from a security agency. After my retirement, my pension salary was inefficient in fulfilling my family needs. With these reasons, I decided to go to another country for the betterment of my family life” (Participant 4).

“After completing my bachelor’s degree, I applied to a lot of government and private jobs, but I was unable to secure a good job. My family financial condition was very bad, due to these reasons, I trusted on a fake and stranger person” (Participant 11).

Likewise, the trafficker’s financial motivation officers also motivated the victims.

“The smuggler was so nice; he gave me a big hope for developing my career and promised me for a good job in Europe with an attractive salary” (Participant 3).

#### Participation of close companions in the recruitment.

Human trafficking is a type of organized criminal activity involving the participation of several persons and organizations that collaborate to carry out the recruitment of victims. Some of the individuals who actively involved in the trafficking process may even be the victims’ close companions, such as their parents, relatives, neighbours or friends. In this study, participants also claimed that their close companions, such as parents, relatives, neighbours, and friends, were involved in their recruitment process.

“The trafficker was an Afghani citizen and had been living in my neighbourhood” (Participant 4).

“The trafficker was my relative” (Participant 6).

“I was introduced to the trafficker by my friend. My friend gave me the contact information of the trafficker” (Participant 13).

“My stepmother was involved in my trafficking process” (Participant 10).

#### The use of cyber platforms in the recruitment .

In the recent past, advancements in information and communication technology such as mobile phones, the Internet, social media applications and many other online networks enabled the traffickers to recruit victims easily. Similarly, the result of this study is based on first-hand victim participants who have also verified the use of online platforms in their recruitment. Many participants clarified that WhatsApp, Facebook, and fake job advertisements play a primary role in their recruitment.

“I came into my first contact with the trafficker, who was in Italy, through Facebook. Subsequently, the trafficker transfers the conversation on WhatsApp. Then he provided me the contact information of his brother, who was in Pakistan and told me to contact him, and he will guide you about further process” (Participant 7).

“My contact was developed through a job advertisement on Facebook; it was a company job in Karachi. They mentioned their WhatsApp number for further conversation” (Participant 11).

### Transportation process

Following the recruitment process, the transportation of victims is the subsequent phase in human trafficking operations. The participants of this research expressed their transportation stories in different manners. Nearly all the participants (n = 12) indicated being transported by traffickers to different locations; two victims were taken domestically, while the remaining eight victims were transported to other countries using different border routes. Moreover, cyber platforms were also utilized in the transportation process. The next two sub-themes provide a more comprehensive explanation of the transportation process.

#### Local transportation used as a means for victim’s transportation.

Local transportation serves as a safe, convenient, economical, and covert means used in several criminal activities. Similarly, the present study participants also verified the same method used by traffickers in their transit. Most of the participants (n = 11) claimed that they were transported using local buses and cars. Likewise, (n = 7) were transported from different locations of KPK, Punjab, and Islamabad towards Quetta, Baluchistan till crossing Taftan (Irani) border.

“The trafficker purchased a bus ticket for me and carried me from Peshawar to Quetta, then from Quetta to Taftan border. All the transportation process was done only using local buses, cars and walking” (Participant 2).

“I went to Lahore from Peshawar, then from Lahore to Karachi, then from Karachi to Quetta through bus. Then in Quetta, they put us in Vigo cars” (Participant 12).

#### Utilization of cyber platforms in the process of transportation.

All the interviewees acknowledged that traffickers had strong networks both domestically and internationally. Traffickers use several technological platforms in the transit process of victims. These technological platforms were utilized for communication, sharing of live locations, and identity sharing (Photos, videos and messages). According to the participants, WhatsApp is the most potent tool which played a crucial role in the transportation process.

“The broker transported me from Peshawar to Quetta. He sent my picture to the Quetta broker on WhatsApp for identification purposes. The broker of Quetta maintained constant communication with me via WhatsApp during the whole journey. He asked me to send the current location time to time. The same process of picture and location was also performed from Quetta to Baluchistan” (Participant 7).

“The trafficker told me to come to Karachi for a job. He sent me the location on WhatsApp. The trafficker was also in contact with me on WhatsApp” (Participant 11).

### Exploitation process

The third most important theme that was revealed from the interviews is the exploitation process. The main motive of the traffickers in human trafficking operations is to withhold the victims under different exploitative conditions. To put it simply, traffickers try to take unfair advantage of the individual victim for forced labor, sexual work, forced marriage, financial gain, and so on. The traffickers use several tactics to accomplish their exploitative aims. In this study, the traffickers also use different strategies during the exploitation of victims. The next two sub-themes describe more comprehensively.

#### Receiving money as a main motive of victim’s exploitation.

Human traffickers’ primary objectives for exploiting victims are revealed by the exploitative circumstances endured by the victims. In this research, the victims were mainly exploited for money gain. For this purpose, the brokers used diverse strategies to extract money from them including forcing the victims into sex, subjecting them to forced labor, making deceptive job offers, and selling and purchasing them.

“Upon arrival in the destination country (Turkey), the trafficker snatched my passport and forced me into working in a hotel for a low wage” (Participant 3).

“I was forced to do massage for men and make customers happy with sex for money purposes. After that, my parents sold me to a forced marriage to gain money” (Participant 10).

“While reaching Iran, the trafficker sold us to another trafficker in Iran… then these traffickers demanded for 100000 PKR per person” (Participant 13).

#### Utilization of cyber platforms in the exploitation process.

Technology is often used by traffickers to exploit their victims. The advancement of technology, namely information and communication networks, offer traffickers additional opportunities to abuse their victims and accomplish their objectives with ease. This study also affirmed, based on the primary data provided by victim participants that technology, including internet, social media applications, making videos and video calls significantly contributed to their victimization.

“They also tortured me using electric current. They removed my clothes and started making my videos for sending to my family on WhatsApp. They sent the account number to my family members and told them to transfer money to this account. They also make video calls to my family for showing my live exploitations. The traffickers released me after they received the money in account” (Participant 4).

“The two females’ traffickers were also sex workers. They also made my videos and pictures and sent these videos and pictures to the customers” (Participant 9).

“I was sexually exploited for six months. They also forced me to make video calls with customers and sell my pictures on social media. It was very hard time for me.” (Participant 10).

### Mental health consequences

The exploitative conditions of human trafficking have led to many victims developing mental health issues. Likewise, the participants in this study faced severe mental health issues after their victimization. Among the mental health consequences, the next two sub-themes presented the participants’ experiences.

#### Adverse psychological implications on victims.

 In this study, in addition to the physical injuries, the victim participants indicated that they faced severe mental health issues such as fear, memory problems, and also suicidal thoughts after their victimization.

“After escaping from traffickers, I was admitted in hospital for almost 4 months. My different body parts were broken. It was very difficult time for me. My sleeping habits changed, and most of the time I felt afraid. I also faced memory problems. I still feel fear when I remember my exploitation time” (Participant 2).

“I was sexually exploited by many men. I cried for many days. I felt like I lost everything in my life. I felt very ashamed and insecure when I memorized the previous movements. My feelings and my mind were destroyed. I also tried to attempt suicide…but my mother gives me a big support” (Participant 9).

#### Adapting behavior towards social media.

Social media technology was mainly used in the overall process of victimization of this study participants. However, the majority of the participants of this study still use and are active on social media, and their behavior towards social media remains unchanged. The participants said that the use of the internet and social media is very important and also economical in today’s society.

“It’s the internet and social media time. It’s very economical to call and make contact on social media. Yes, I am carefully using social media, but it’s impossible to leave it” (Participant 5).

“The use of social media is very important for our daily life; I have no issue on social media” (Participant 12).

## Discussion

This study attempted to concisely examine the role of technology in human trafficking activities, based on the firsthand experiences of victims from two provinces of Pakistan. Four main themes, namely the recruitment process, transportation process, exploitation process and mental health consequences were obtained via interviews with the victims.

The first theme consists of four sub-themes, namely, the onset of victimization at a younger age, financial motivation is a leading factor of recruitment, participation of close companions in the recruitment, and involvement of cyber platforms in the recruitment. The victims of human trafficking were mainly recruited and exploited at a younger age. Several studies confirmed that traffickers mostly preyed on the young demographic. For example, young people who are struggling for better jobs and life styles are the ones that traffickers target and ensnare [51–53]. The traffickers attempt to get victims into the trafficking business by forming amorous bonds and romantic relationships [54–57]. In addition, several factors are making a major contribution in the recruitment of victims. However, the results of this study indicated that financial motivation is an essential factor in the recruitment of victims. Other studies have also verified the contribution of this factor in the victim’s recruitment. For example, a study conducted on a sample of 249 young individuals in New York revealed that financial motivation is a leading factor for involving them into sex trafficking [58]. During these operations, the traffickers mostly entice the victims by offering better opportunities, job offers, and hope for a better life [59–62]. The UNODC 2020 report disclosed that the economic need is the major factor in 51% of human trafficking cases. Consequently, the recruitment of victims is facilitated by the involvement of several actors [33,63]. Many studies have also documented that close companions, such as family members, husband, brother, mother, sister, neighbors and friends provide a basic contribution to the recruitment of victims [62,64,65]. Simultaneously, the victim recruiting strategies undergo a transition from conventional approaches to innovative cyber techniques. The traffickers use the internet and social media platforms in the recruitment and trapping of victims [66,67]. Many other studies have also shown that social media platforms, particularly Facebook, and Instagram are used for the dissemination of bogus job advertisements and creation of phony accounts to target potential victims [26,68–70].

Transportation process is the second main theme. This study’s results articulated that local transportation was used as the main method for victim transportation. Most of the victims had traveled between different locations using local buses and cars. Likewise, many previous studies also affirmed that traffickers used the local transportation for victims’ movement. The IBM Traffic Analysis Hub asserted that more than 3,019 human trafficking cases documented in the US between 2020 and 2021. In approximately 2,400 of these cases, local transportation methods such as buses, cars and trucks were used [71]. Teshome et al., 2018 found that majority of Ethiopians were trafficked to Yemen by local vehicles, including local cars, trucks and containers [72]. The traffickers have also strong connections both domestically and internationally. The victims of this study said that traffickers used internet and WhatsApp application to maintain constant communication throughout the transit procedure. To ensure continuous contact and tracing locations, the traffickers extensively use the social media networks, such as internet, mobile phones and WhatsApp in the transportation process [73,74]. In this regard, a study conducted in the UK found that internet, mobile phones, WhatsApp, and Viber are often used for coordinating victims transportation, communication, and sharing locations to facilitate their entry into the UK [75].

After the recruitment and transportation procedures, the interviews with the victims highlighted the exploitation process as the third most significant theme. The traffickers primarily engaged in the trafficking of individuals with the intention of exploiting them, using different strategies to execute their objectives [76]. The victims of this research confirmed that the main goal of traffickers was to get financial advantage. Hence, the traffickers performed several strategies such as offering fake jobs, coercing victims into low-wage labor, buying and selling, and compel them to engage in sex work, all with the aim of attaining their financial goals. Other studies have also recorded that traffickers exploit the victims for sex slavery, forced marriages, forced labor, and buying and selling for profit [76]. Following this, it was documented that human trafficking industry has generated more than 150US billion dollars annually. The traffickers generated money from victims sexual exploitation, forced labor, violence, and threats against both victims and their families [77–79]. On the other hand, traffickers also use different cyber platforms to efficiently and expeditiously accomplish their goals [68]. This study asserted that the victims were extensively exploited via the use of internet, social media apps, live streaming, and video calls. The victims further detailed that traffickers use video calls and transmit their exploitative recordings to their relatives as a means of intimidation and extortion and demanding money. According to Mohamad et al., in many cases, the traffickers use the strategies of videos, taking pictures of the victims in order to exploit and control them [73]. A recent 2021 UNODC Report also affirmed that traffickers extract control over their victims by making their videos and send to their families in order to achieve their objectives [80].

The next main theme explored from the interviews was the mental health consequences. The activities of human trafficking put several victims in huge physical and psychological consequences. All the participants in this research experienced negative psychological effects, such as dread, memory problems, nightmares, and suicidal thoughts. Similarly, a study on women and girls trafficked for forced marriages in Pakistan had also observed that the majority of women and girls experienced severe physical abuse, mental disorders, violence, and traumatic situations [81]. In addition, a research carried out in Nigeria revealed that over 90% of trafficked individuals experienced severe torture, sexual assault, despair, and threatening circumstances [82]. Another study in England reported that 78% of female victims survivors and 40% of male victims survivors were diagnosed with various psychiatric problems [83]. In addition to this, the current study had also explored the adaptation behaviours that the victims had engaged in, in terms of their social media usage after their experience of being trafficked. Several victims indicated that their behavior towards social media remains unchanged. The victims emphasized that the usage of social media is important for our daily life, and it is also a cheaper way to stay in contact with domestic or international contacts.

## Limitation and advantages

This study was the first of its kind to extensively address the role of technology in the process of human trafficking victimization from the lived experiences of victims in Pakistan. The findings of this study unveiled the concealed and most important aspects of technology that widely contributed to the recruitment, transportation, and exploitation of victims. The next advantage of this qualitative study was the participation of victim participants from different geographical locations, and also the contribution of both male and female victims. In addition, the findings of this study will be useful for the academic scholars, researchers and other relevant institutional stakeholders, including law enforcement agencies, legislative bodies, policy makers and civil society, from Pakistan and worldwide. However, this study also has several limitations. Since the victims of human trafficking are the hidden population in the society [84]. Due to the sensitive nature of the topic, securing an ethical approval code was not easy. However, the study was approved by the university ethical committee due to the importance and implications of the study. It was difficult to access the participants. Therefore, the interview sessions were only conducted once with each of the participants. Purposive and snowball sampling methods were adopted in this study to recruit the participants. Lastly, this study was limited to only two provinces of Pakistan.

## Conclusion

Human trafficking is seeing a significant surge on a global scale, affecting both developed and less developed nations, including Pakistan. Technological advancements, namely internet and social media, have opened a new kind of development in human trafficking industry [13]. In this regard, the present qualitative study explored a number of important themes and sub-themes that open up new avenues for future qualitative and quantitative research under technology and human trafficking jurisdiction, both in developed and less developed countries. The findings of this study indicated that traffickers extensively use technology, including the internet, mobile phones, and social media apps, across all phases of human trafficking, encompassing the identification, recruitment, transportation, and exploitation of victims. Furthermore, it has also been identified that the online exploitation tactics have significant physical and psychological consequences on the victims. In parallel, the unique contribution of this study is that it provides an open academic platform to victims’ voices with regards to their victimization. This qualitative research is expected to open the doors for future studies and will also provide a valuable contribution to the law enforcement agencies, legislative bodies, policymakers, civil society and SDG (2030) goals, particularly 8.7, 5.2, 16.2, 17.6, in Pakistan and globally, in their efforts to address and enhance the learning and awareness levels regarding websites, applications and other related technologies in order to improve investigation in combating technological based human trafficking.

## Supporting information

S1 File Inclusivity-in-global-research-questionnaire. (DOCX)

S2 File Victims interview guide. (DOCX)
