# Supplementary material for: Exploring the role of technology in human trafficking in Pakistan: A qualitative study of lived experiences of victims
Source: PLoS One. 2025 Mar 25;20(3):e0320088. doi: 10.1371/journal.pone.0320088 (PMC11936256; doi:10.1371/journal.pone.0320088)
Supplement: S2 File — (DOCX) [file pone.0320088.s002.docx]

**Interview Guide for Victims متاثرین کے لئے انٹرویو گائیڈ**

| **S/N** | **Themes موضوعات** | **Probing** تحقیقات |
| --- | --- | --- |
| 1 | Please tell me about yourself  براہ مہربانی مجھے اپنے بارے میں بتائیں | (i) Gender (ii) Age (iii) Marital status  (iv) Educational level (v) Current place of living  (vi) Occupational status (current and past)  (viii) Your family monthly income including all resources.  جنس (١)  عمر (٢)  ازدواجی حیثیت(٣)  تعلیمی قابلیت(٤)  موجودہ رہائش گاہ(٥)  پیشہ ورانہ حیثیت (موجودہ اور ماضی)(٦)  آپ کے خاندان کی ماہانہ آمدنی جس میں تمام وسائل شامل ہیں(٧) |
| 2 | Please tell me about your social media and computer technology usage  براہ مہربانی مجھے اپنے سوشل میڈیا اور کمپیوٹر ٹیکنالوجی کے استعمال کے بارے میں بتائیں | (i) Type of mobile usage (ii) Computer use  (iii) Internet use (iv) Number of applications  (v) Fake/real account (vi) Dependency on usage  (vii) How much and why you use.  موبائل استعمال کی قسم(١)  کمپیوٹر کا استعمال(٢)  انٹرنیٹ کا استعمال(٣)  ایپلی کیشنز کی تعداد(٤)  جعلی / حقیقی اکاؤنٹ(٥)  استعمال پر انحصار(٦)  آپ کتنا اور کیوں استعمال کرتے ہیں(٧) |
| 3 | What age were you when you fell prey to cyber-based human trafficking?  جب آپ سائبر پر مبنی انسانی سوداگری کا شکار ہوئے تو آپ کی عمر کیا تھی؟ |  |
| 4 | Please explain how you were being victimized through using online cyber platform.  برائے مہربانی وضاحت کریں کہ آن لائن سائبر پلیٹ فارم کا استعمال کرتے ہوئے آپ کو کس طرح نشانہ بنایا جارہا تھا۔ | (i) Which cyber platform were used  (ii) Who were involved in the process  (iii) What commitment did they make at first  (iv) What type of language they use  (v) What type of words they use  (vi) His/her profile picture.  کون سا سائبر پلیٹ فارم استعمال کیا گیا۔(١)  اس عمل میں کون شامل تھا(٢)  انہوں نے پہلے کیا وعدہ کیا تھا(٣)  وہ کس قسم کی زبان استعمال کرتے تھے(٤)  وہ کس قسم کے الفاظ استعمال کر رہے تھے(٥)  اس کی پروفائل تصویر(٦) |
| 5 | What reasons led you to the victimization by using online cyber platform?  کون سی وجوہات آپ کو آن لائن سائبر پلیٹ فارم کا استعمال کرکے استحصال کی طرف لے گئیں؟ | (i) Better life (ii) Better offers by traffickers  (iii) Unemployment  (iv) Family’s financial condition/needs or any other reason.  بہتر زندگی(١)  سوداگروں کی جانب سے بہتر پیشکشیں۔(٢)  بے روزگاری۔(٣)  خاندان کی بری حالت یا کوئی اور(٤) |
| 6 | Did you have any doubts about the trafficker during your initial online communication? If yes, please explain why. If not, please explain the reasons.  کیا آپ کو اپنے ابتدائی آن لائن مواصلات کے دوران سوداگر کے بارے میں کوئی شک تھا ؟ اگر ہاں، تو براہ مہربانی وضاحت کریں کیوں. اگر نہیں، تو براہ مہربانی وجوہات کی وضاحت کریں. |  |
| 7 | How you transited from one place to another place? Explain the process  آپ ایک جگہ سے دوسری جگہ کیسے منتقل ہوئے؟ عمل کی وضاحت کریں | (i) What cyber platforms were used during this phase  (ii) How did they use  (iii) What tactics traffickers employed.  (١)اس مرحلے کے دوران کون سے سائبر پلیٹ فارم استعمال کیے گئے۔  انہوں نے کس طرح استعمال کیا(٢)  سوداگروں نے کیا ہتھکنڈے استعمال کیے(٣) |
| 8 | Did they exploit you by using any technological tool during transactions from one place to another place? If yes, please explain why and how? If not, please explain the reasons?  کیا انہوں نے ایک جگہ سے دوسری جگہ منتقلی کے دوران  کسی تکنیکی آلے کا استعمال کرکے آپ کا استحصال کیا؟ اگر ہاں، تو براہ مہربانی وضاحت کریں کیوں اور کیسے؟ اگر نہیں، تو براہ مہربانی وجوہات کی وضاحت کریں؟ | (i) What cyber platforms were used  (ii) They make your videos, pictures  (iii) Sexually, physically or psychologically tortured you.  کون سے سائبر پلیٹ فارم استعمال کیے گئے (١)  وہ آپ کی ویڈیوز، تصاویر بنا رہے تھے(٢)  آپ کو جنسی، جسمانی یا نفسیاتی طور پر تشدد کا نشانہ بنایا گیا(٣) |
| 9 | After you arrived at the destination point, for what purpose traffickers exploited you? Please explain  منزل مقصود پر پہنچنے کے بعد سوداگروں نے کس مقصد کے لیے آپ کا استحصال کیا؟ براہ مہربانی وضاحت کریں | (i) Sexual (ii) labor (iii) Forced marriage  (iv) Financial gain (v) Online sale purchase  (vi) Making your videos or pictures  (vii) Advertised you on social media etc.  جنسی(١)  کام کرنا(٢)  جبری شادی(٣)  مالی فائدہ(٤)  آن لائن فروخت کی خریداری(٥)  آپکی ویڈیوز یا تصاویر بنائیں(٦)  سوشل میڈیا وغیرہ پر آپ کی تشہیر کی(٧) |
| 10 | Did traffickers threaten your family members or demanding for money while using any technological tools? If yes, please explain why. If not, please explain the reasons.  کیا سوداگروں نے کسی بھی تکنیکی اوزار کا استعمال کرتے ہوئے آپ کے خاندان کے ممبروں کو دھمکی دی یا پیسے کا مطالبہ کیا؟ اگر ہاں، تو براہ مہربانی وضاحت کریں کیوں. اگر نہیں، تو براہ مہربانی وجوہات کی وضاحت کریں | (i) Calling or message your family  (ii) Send your videos and pictures to your family etc.  آپکی خاندان کو کال کرنا یا پیغام بھیجنا(١)  آپکی ویڈیوز اور تصاویر اپنے خاندان وغیرہ کے لئے بھیجیں(٢) |
| 11 | After being victimized, what social and psychological implications are you facing on social media? Please explain  تشدد کا نشانہ بننے کے بعد، آپ کو سوشل میڈیا پر کس سماجی اور نفسیاتی مضمرات کا سامنا کرنا پڑ رہا ہے؟ براہ مہربانی وضاحت کریں | (i) Feeling insecure on social media  (ii) Still threats received from traffickers  (iii) Mental disturbance  (iv) Attempted suicide or ideation/thoughts  (v) Stigmatization from your family members or others in the society.  سوشل میڈیا پر غیر محفوظ محسوس کر رہے ہیں،(١)  اب بھی سوداگروں کی جانب سے دھمکیاں مل رہی ہیں۔(٢)  ذہنی اضطراب۔(٣)  خودکشی کرنے کی کوشش یا خیالات(٤)  آپ کے خاندان کے ممبروں یا معاشرے کی طرف سے بدنامی(٥) |
